# Parallel analysis and MBI-HSS: How many factors?

**Published:** 2013-12-31

**Authors:** Cesar Merino Soto, Marisol Angulo Ramos

## Mr. Editor

It has been only recently possible to validate the *Maslach Burnout Inventory-Human Services Survey* (MBI-HSS)^1^ among health professionals of Cali^2^, an important step for using this instrument with local empirical support in regard to its reliability of scoring and internal structure. However, two aspects of this analysis can be considered as methodological weaknesses. First, the Cronbach alpha coefficient was calculated for the total group of items, and this is absolutely inappropriate because: a)the authors did not demonstrate empirical support for accomplishing this (e.g., a hierarchical factor analysis), b) the literature indicates that factors in the MBI-HSS are generally independent, a characteristic also reported by Córdoba *et al*.^2^,and the same authors of the MBI-HSS^1^) the authors did not report the inter-factor correlations with which an appreciation could have been obtained, at least an heuristic one of the common degree of variance among the factors.Secondly, the authors obtained seven factors in their exploratory factor analysis; this large number of actors seems to be a product of applying a factor retention method that is now consensually seen as inaccurate and little recommended^3^ ^,^ ^4^ ^. ^Specifically, it is known as *Kaiser's rule*, *Guttman´s rule* or *simply K1* ^4^. The problem identified with this method is its over-estimation of the number of factors to be retained^3^ ^,^ ^4^, a situation that clearly occurs in the results reported by Cordoba *et al*.^2^, as reported in their Table 2.

A more accurate method which has gained a scientific consensus for good practices for retaining the number of factors is called *parallel analysis* ^3^ ^,^ ^4^. This procedure is based on the work of Horn^4^, which consists of randomly creating the same number of variables as the number of items analyzed (in the case of MBI-HSS, 22 items),correlating them and extracting eigenvalues​​ against which the eigenvalues derived from the empirical data under analysis are compared. This procedure was applied to the eigenvalues ​​reported by Cordoba *et al.* ^2^, in Table 2 by means of the *Monte Carlo PCA* ^5^ program (100 replications). Our results are shown in (Table 1).

**Table 1:**
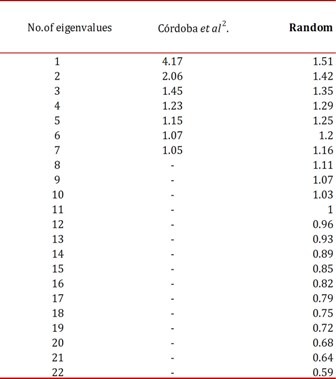
The eigenvalues of Cordoba et al and those generated randomly (100 replications)

The appropriate number of factors to retain is achieved by making a one to one comparisons of the eigenvalues, keeping the empirical eigenvalue that is less than the random eigenvalue. Conceptually, this indicates that the significant eigenvalues ​​must be greater than those generated randomly. In Table 1, the number of eigenvalues ​​to retain is 3, which is exactly the same number of factors underlying the MBI-HSS. Validating the result with a conceptual analysis of these three factors, would leave one to conclude that the factorial structure of the MBI-HSS is replicable in the sample studied. Compared with the first result of the authors (7 factors), the methodological difference is clear. Finally, we note that the use of parallel analysis should be recommended for making more accurate decisions about the number of factors to retain.
